# Supplementary material for: Blue light-emitting diodes based on colloidal quantum dots with reduced surface-bulk coupling
Source: Nat Commun. 2023 Jan 17;14:284. doi: 10.1038/s41467-023-35954-x (PMC9845229; doi:10.1038/s41467-023-35954-x)
Supplement: Supplementary file 1 — Supplementary Information [file 41467_2023_35954_MOESM1_ESM.pdf]

Supplementary Information

Blue Light-Emitting Diodes Based on Colloidal Quantum Dots with Reduced Surface-Bulk Coupling

Xingtong Chen[1,2], Xiongfeng Lin[3], Likuan Zhou[3], Xiaojuan Sun[1,2], Rui Li[1,2], Mengyu Chen[1,2], Yixing Yang[3], Wenjun Hou[3], Longjia Wu[3]*, Weiran Cao[3], Xin Zhang[3], Xiaolin Yan[3], and Song Chen[1,2]*

[1] Suzhou Key Laboratory of Novel Semiconductor-optoelectronics Materials and Devices, College of Chemistry, Chemical Engineering and Materials Science, Soochow University, Suzhou 215123, Jiangsu, China.

[2] Jiangsu Key Laboratory of Advanced Negative Carbon Technologies, Soochow University, Suzhou, 215123, Jiangsu, PR China

[3] TCL Corporate Research, 1001 Zhongshan Park Road, Nanshan District, Shenzhen 518067, Guangdong, China

Correspondence and requests for materials should be addressed to S.C. (email: songchen@suda.edu.cn) or to L.W. (email: wulongjia@tcl.com)

Table of Contents

1. **Supplementary Note**

   Supplementary Note 1

2. **Supplementary Figure**

   Supplementary Figures 1-27

3. **Supplementary Table**

   Supplementary Table 1

4. **Supplementary References**

   Supplementary References 1-19

Supplementary Note 1

Derivation of fitting equation for the model of EA:

Fitting the EA spectra of the pure QD film using Equation 1 generates the dipole moment change between the 1st excited state and the ground state ($\Delta\mu$). Due to the nonpolar nature of the s-orbital-based ground state, $\Delta\mu$ reflects the dipole moment of the excited state. In the Stark effect, the dipole interacts with an applied electric field, leading to the Stark shift in transition energy (ΔE) of an excited state. L. Sebastain et al[1] proposed that ΔE is defined as the difference between the shift of the excited state energy and the shift of the ground state energy:

$$\Delta E = -\Delta\mu_z \cdot F - \frac{1}{2}\Delta p \cdot F^2 \quad (1)$$

where Δp is the change in the average polarizability tensor upon excitation, $\Delta\mu_z$ is the difference between the ground and excited-state dipole moments, and F is the strength of the electric field.

EA spectra record the change in the absorption coefficient ($\Delta\alpha$) under an electric field. The fitting equation of electroabsorption can be correlated with the Stark effect and measured light intensity. The perturbation level absorption changes ($\Delta\alpha$) can be expressed in terms of the McLaurin series truncated at the quadratic term:

$$\Delta\alpha \approx \Delta E\frac{\partial\alpha}{\partial E} + \frac{1}{2}(\Delta E)^2\frac{\partial^2\alpha}{\partial E^2} \ldots \quad (2)$$

Combining Equations 1 and 2 yields the relationship between $\Delta\alpha$ and the derivatives of α:

$$\Delta\alpha \approx \left(-\Delta\mu_z F - \frac{1}{2}\Delta p F^2\right)\frac{\partial\alpha}{\partial E} + \frac{1}{2}\left(-\Delta\mu_z F - \frac{1}{2}\Delta p F^2\right)^2\frac{\partial^2\alpha}{\partial E^2} \quad (3)$$

It is noteworthy that Equation (2) is only meaningful in describing the change in the absorption coefficient of a single quantum dot. For the thin films, the change in the absorption coefficient is an isotropic average over all orientations, and therefore, the change in the dipole moment along the electrical field can be positive or negative with equal probability. Thus, the term that contains $\Delta\mu_z$ in the first bracket of Equation (3) vanishes. For the second bracket in Equation (3), we approximate the second-order dependence of the electrical field. Meanwhile, the dipole moment in any dimension is

the same in magnitude; thus:

$$\Delta\mu_x{}^2 = \Delta\mu_y{}^2 = \Delta\mu_z{}^2 = \frac{1}{3}\Delta\mu^2 \tag{4}$$

Now, $\Delta\mu$ in Equation (4) is the macroscopic average value of the permanent dipole moment change for all orientations. Here, by applying Equation (4) to Equation (3), the absorption coefficient ($\Delta\alpha$) changes can be expressed as[2,3]:

$$\Delta\alpha \approx \frac{1}{2}\Delta p F^2 \frac{\partial\alpha}{\partial E} + \frac{1}{6}\Delta\mu^2 F^2 \frac{\partial^2\alpha}{\partial E^2} \tag{5}$$

Considering the relationship between the absorption coefficient and absorbance A[4], Equation (5) can be further expressed as:

$$\Delta\alpha \approx \frac{1}{0.86}\Delta p F^2 \frac{\partial A}{D\partial E} + \frac{1}{2.58}\Delta\mu^2 F^2 \frac{\partial^2 A}{D\partial E^2} \tag{6}$$

D is the thickness of the active layer. Finally, we can get the fitting equation:

$$-\frac{\Delta T}{T} = \Delta\alpha \cdot d \approx \frac{1}{0.86}\Delta p F^2 \frac{d\partial A}{D\partial E} + \frac{1}{2.58}\Delta\mu^2 F^2 \frac{d\partial^2 A}{D\partial E^2} \tag{7}$$

where the film thickness is approximate to the optical path length in transmission mode[5]. Therefore, $\Delta\alpha$ can be explicitly expressed as a function of changes in the average polarizability Δp and average permanent dipole moment $\Delta\mu^2$ of the material upon photoexcitation from the ground state to the excited state. $\frac{\partial A}{\partial E}$ and $\frac{\partial^2 A}{\partial E^2}$ are the 1st and 2nd derivatives of the optical absorption of the material, and F is the electrical field.

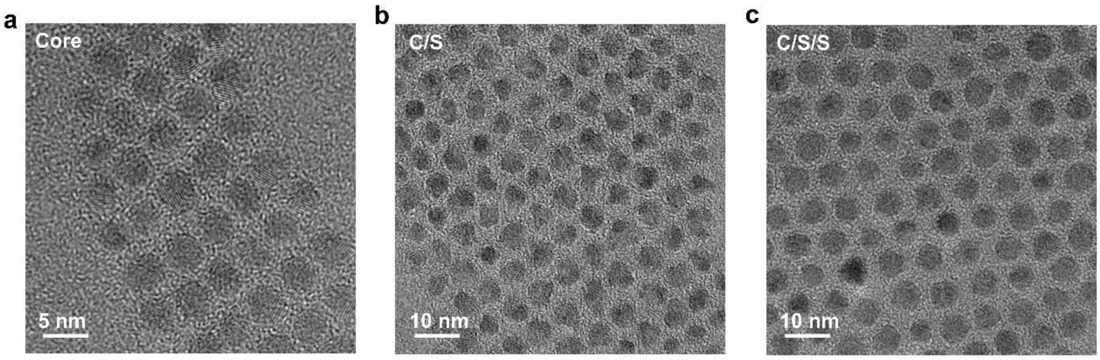

Supplementary Fig. 1 | TEM images of Q1. a-c, ZnCdSe(core), ZnCdSe/ZnCdSeS(C/S) and ZnCdSe/ZnCdSeS/ZnS (C/S/S) quantum dots, respectively.

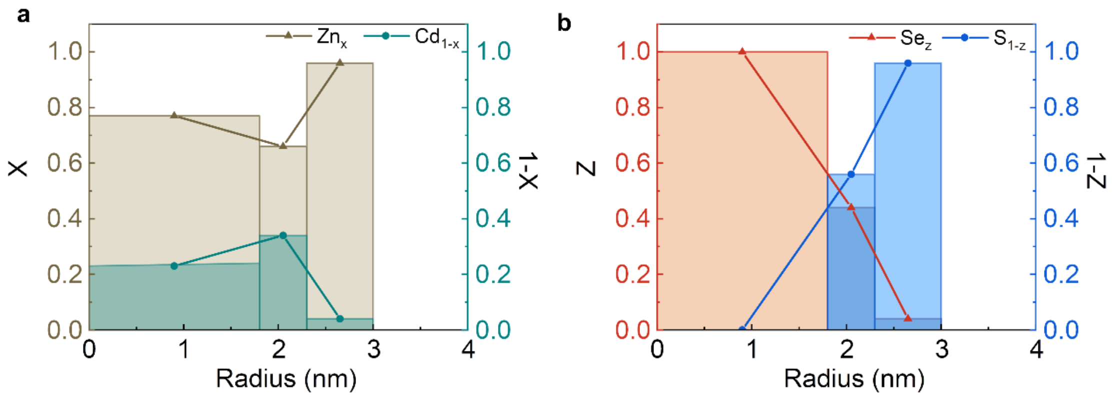

Supplementary Fig. 2 | The compositional profile in the radial direction of Q1 ($Zn_xCd_{1-x}Se_z/Zn_xCd_{1-x}Se_zS_{1-z}/Zn_xS_{1-z}$)**. a,** Radial distribution of cations. **b,** Radial distribution of anions. The results were obtained by measuring ICP-OES and TEM.

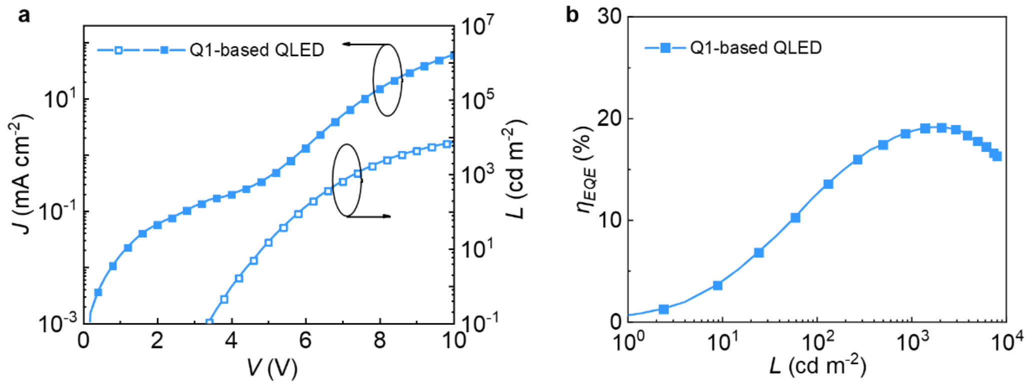

Supplementary Fig. 3 | Device performances of Q1-based QLEDs. a, *L*-*J*-*V* characteristics. **b,** External quantum efficiencies (EQEs).

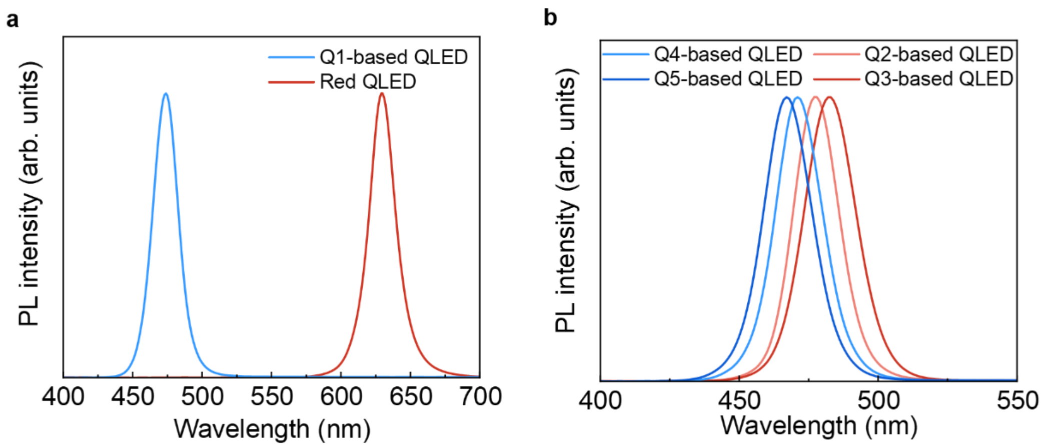

Supplementary Fig. 4 | Normalized electroluminescence spectra. a, Red and Q1-based QLEDs. **b,** Q2-, Q3-, Q4-, and Q5-based QLEDs.

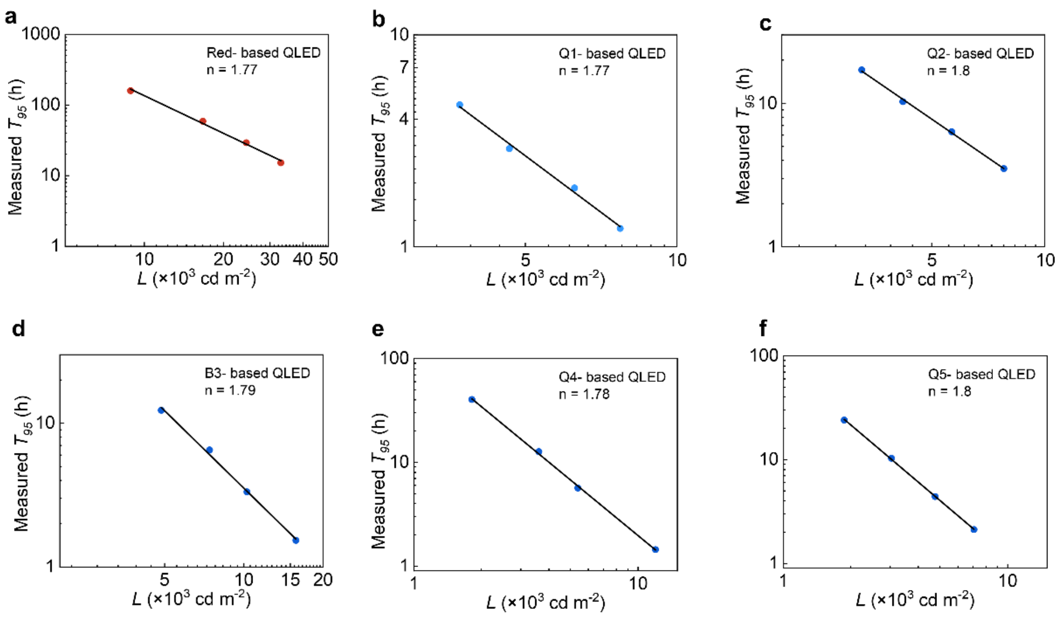

Supplementary Fig. 5 | Experimentally determined acceleration factor. a-f, Operational lifetime of red, Q1-, Q2-, Q3-, Q4- and Q5-based QLEDs measured at different initial brightness, respectively. The data are fitted by an empirical equation, $L_0^n \times T_{95}$ = constant so as to generate the acceleration factors.

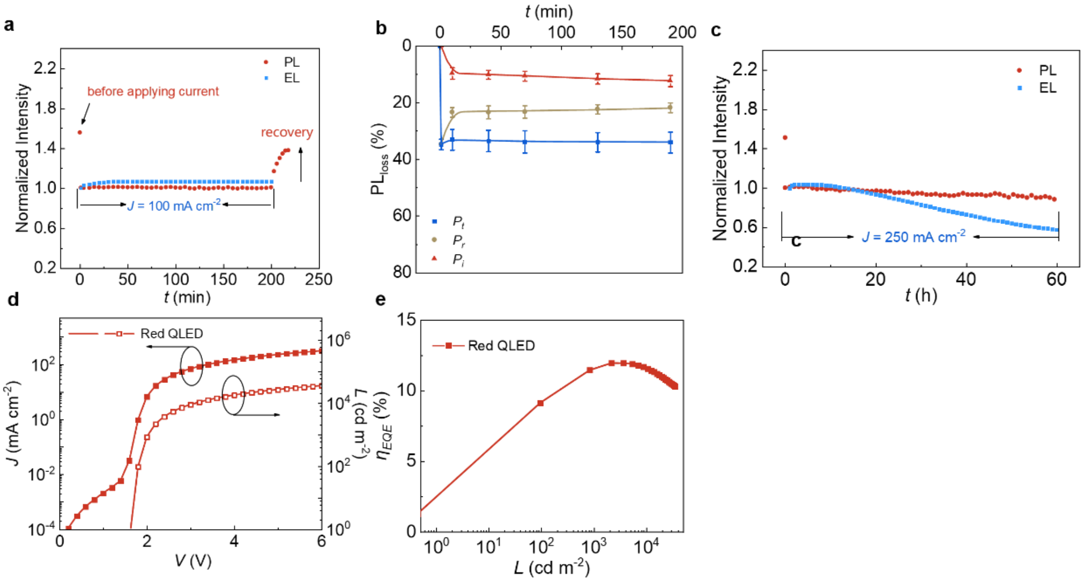

Supplementary Fig. 6 | Device performances of red QLEDs. a, PL and EL intensity of red QLEDs monitored as a function of operation time. **b,** Reversible, irreversible and total PL loss of red measured from corresponding QLEDs as a function of device operation time. The tests are done using constant current density of 100 mA cm^{-2}. The error bars are the standard deviations calculated from the same sample. **c,** Simultaneous monitoring of EL-PL from the same red QLED as a function of operation time. **d,** *L-J-V* characteristics. **e,** External quantum efficiencies (EQEs) and luminance efficiency.

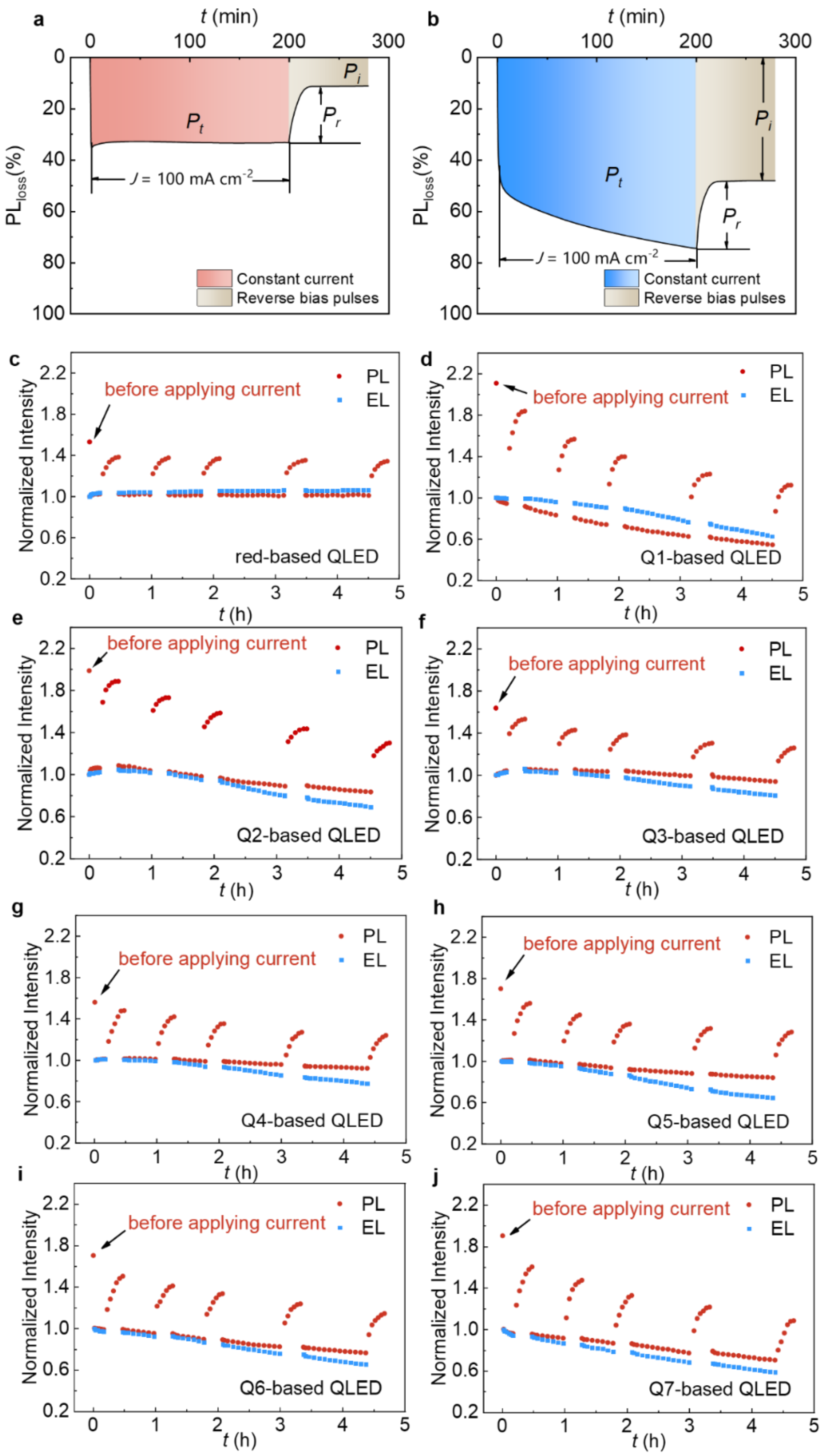

Supplementary Fig. 7 | The reversible and irreversible degradation of blue QDs. **a and b,** Temporal change of PL measured for red and Q1-based QLED, respectively. P_t, P_r, and P_i represent the total PL loss, the reversible degradation, and the irreversible degradation of QDs ($P_t = P_r + P_i$), respectively. **c-j,** Temporal P_t, P_r and P_i values of red, Q1-, Q2-, Q3-, Q4-, Q5-, Q6-, and Q7-based QLEDs, respectively. The current density is 100 mA cm^{-2} during all the tests. The recovery of PL was monitored after each interruption in which the forward current source was immediately switched to continuous reverse bias pulses.

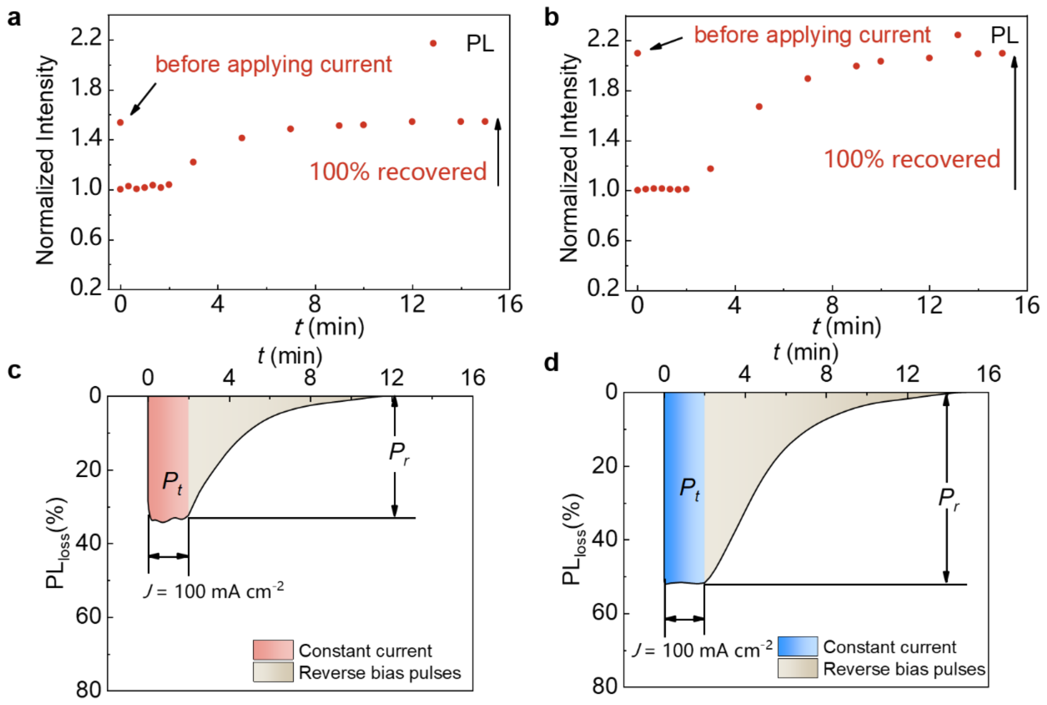

Supplementary Fig. 8 | Complete recovery of PL loss. a and b, Results of EL-PL test of red and Q1-based QLEDs. **c and d,** PL recovery for red and Q1-based QLEDs after an operation of 2 min, respectively. The PL recovery is 100% ($P_i = 0$, $P_t = P_r$).

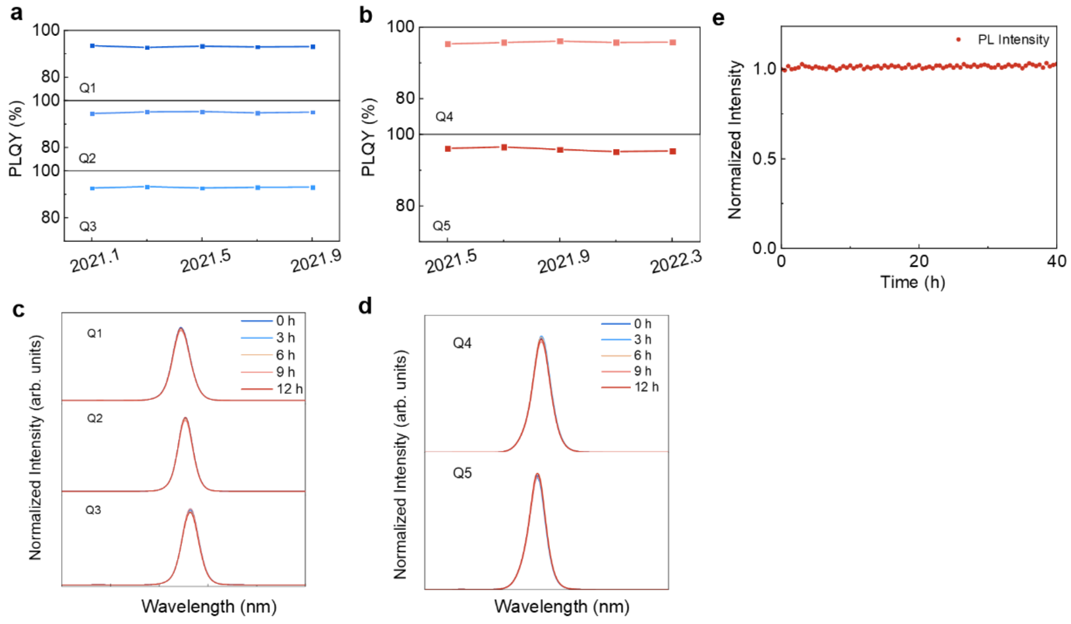

Supplementary Fig. 9 | Photoluminescence stability, a and b, Shelf stability of Q1-Q5; **c and d,** Thermal stability of Q1-Q5. The PL spectra were taken by were measured when QD was continuously heated at 90 ºC. These QDs show high intrinsic stabilities upon storage over 270 days and continuous heating by 12 h. **e,** Q4's photoluminescence under continuous excitation (2.5 mW cm^{-2}), indicating ultrahigh intrinsic stability.

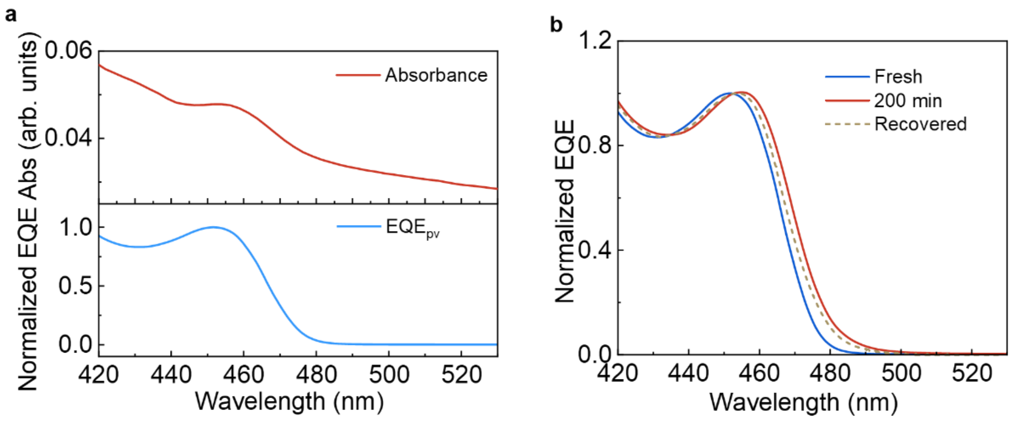

Supplementary Fig. 10 | Sensitive external quantum efficiency spectrum vs. plain absorption. a, Upper: Optical absorption spectra of a QD film. Below: Spectra of sensitive external quantum efficiency measured for a QLED comprising the same batch of QDs in the photovoltaic mode. **b,** The redshift of the excitonic transition (22.6 meV) due to device operation and partial recovery (6.6 meV) after applying a reverse bias. The constant current density is set at 100 mA cm^{-2}.

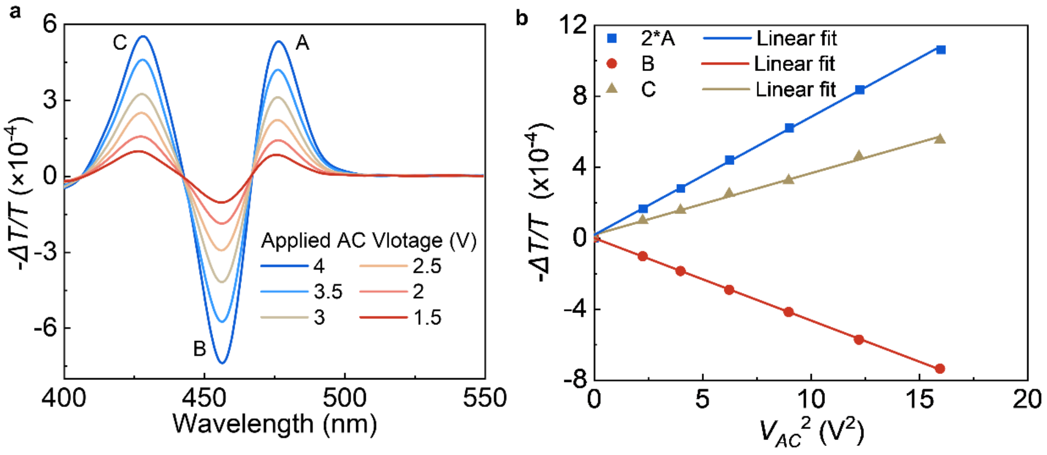

Supplementary Fig. 11 | Field dependence of the Stark Effect. a, EA spectra of Q1-based QLEDs under variant AC voltages and DC voltage was fixed at -3 V. **b,** The amplitude of A, B and C plotted as a function of the square of AC voltages (V_{AC}^2). The linear dependence confirms the EA signals origin from the Quantum Confined Stark effect.

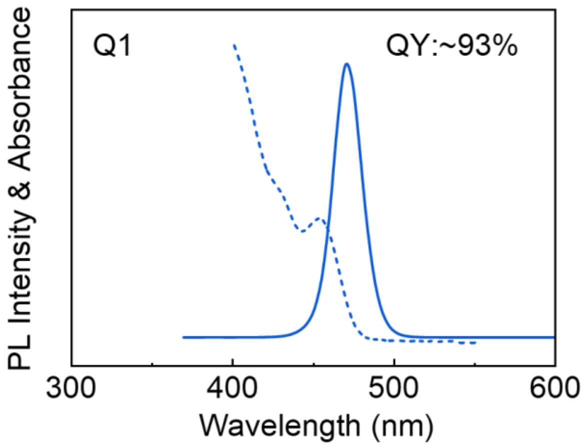

Supplementary Fig. 12 | PL spectra and absorbance spectra of Q1.

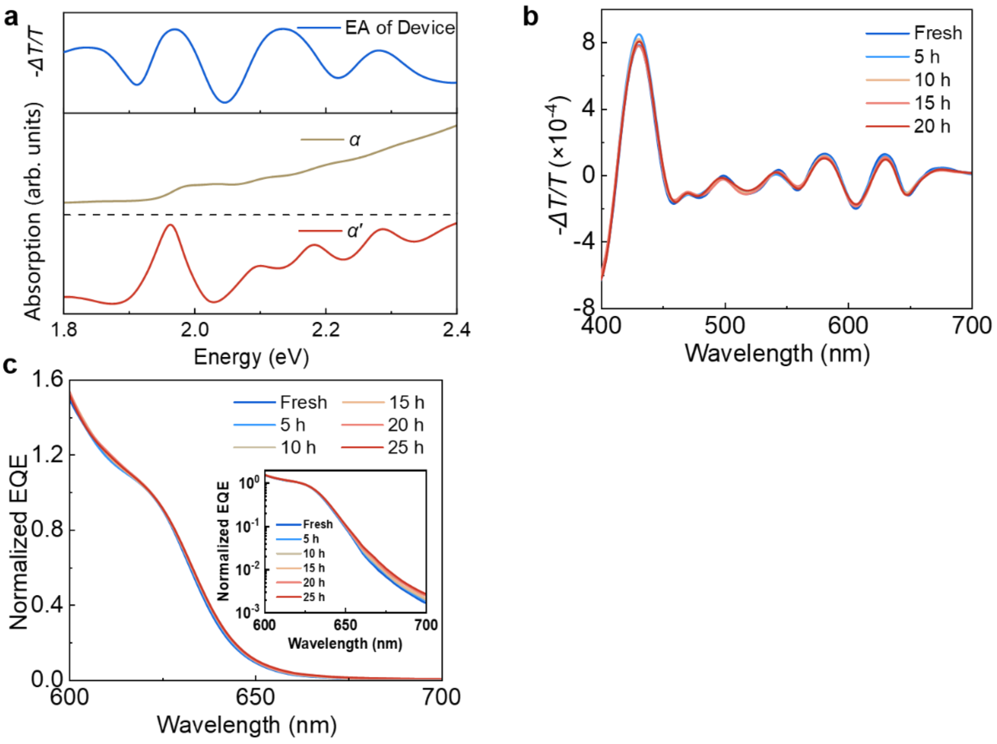

Supplementary Fig. 13 | The nonpolar excited state of red QDs. a, Upper: Electroabsorption spectra of red-based QLED. Below: the lineshape of absorption coefficient (α) and first derivative of α (α') of red QDs. **b,** Electroabsorption spectra of red QLEDs, which were driven using a constant current density of 250 mA cm^{-2} for different length of time. Notably, the EA spectra remain almost unchanged even after an operation for 20 h. **c**, sEQE$_{PV}$ spectra of red based QLEDs. The inset shows the same sEQE$_{PV}$ plotted on a logarithmic scale for the demonstration of the near-band-edge transitions.

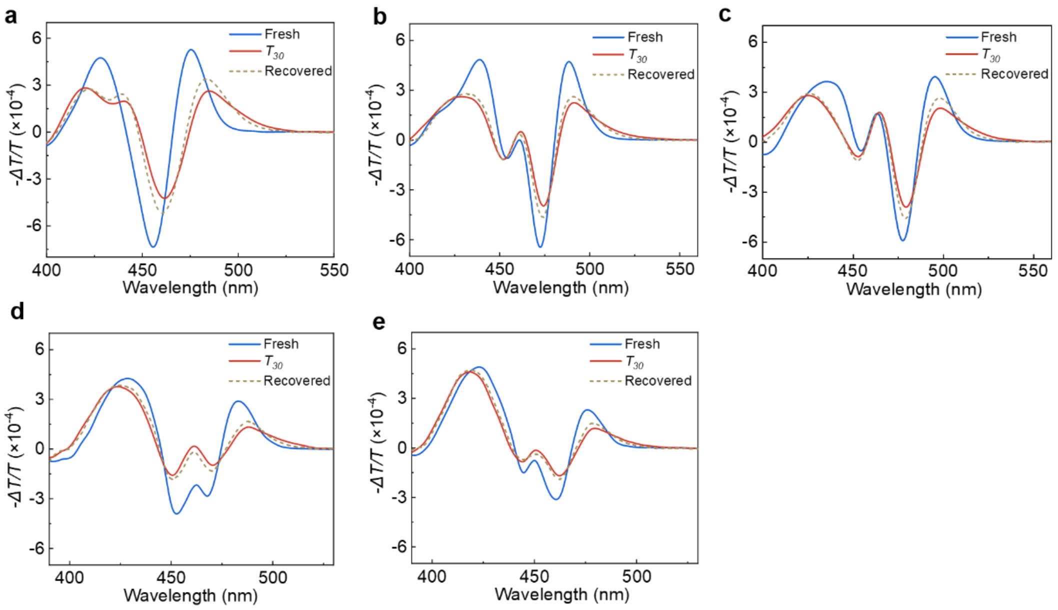

Supplementary Fig. 14 | EA spectral change and partial recovery. a-e, EA spectra of Q1-, Q2-, Q3-, Q4- and Q5-based QLEDs, respectively. The measurements were carried out when the devices were freshly made, degraded to T_{30}, and recovered by reverse bias.

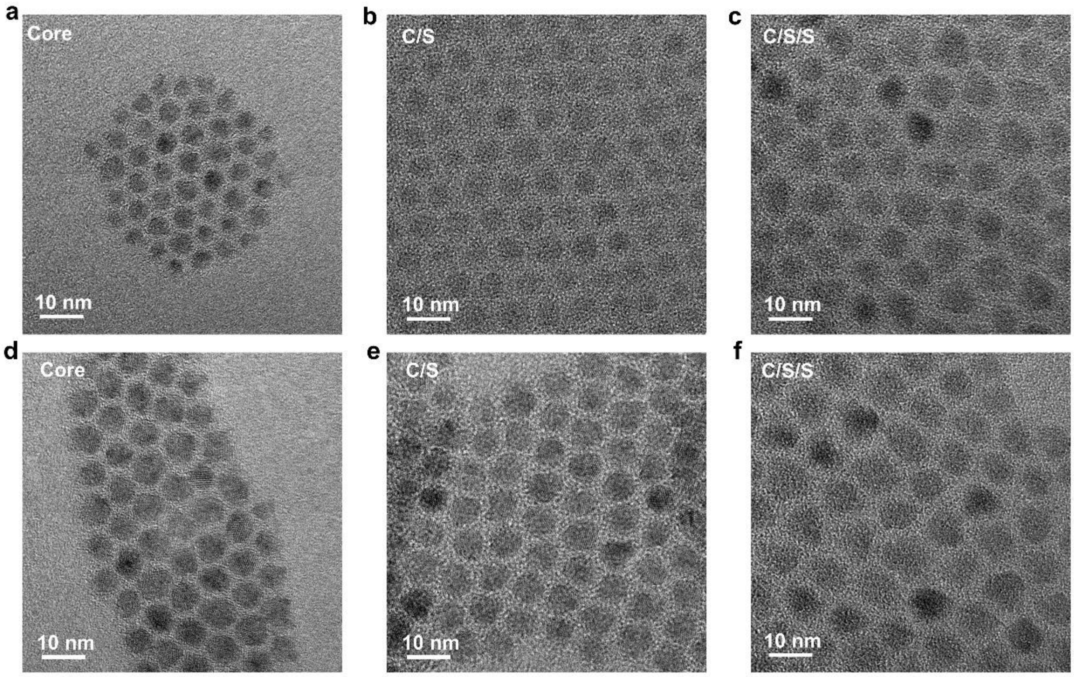

Supplementary Fig. 15 | TEM images of Q2 and Q3. a-c, ZnCdSe (core), ZnCdSe/ZnCdSeS (C/S), and ZnCdSe/ZnCdSeS/ZnS (C/S/S) of Q2, respectively. **d-f,** ZnCdSe (core), ZnCdSe/ZnCdSeS (C/S), and ZnCdSe/ZnCdSeS/ZnS (C/S/S) of Q3, respectively.

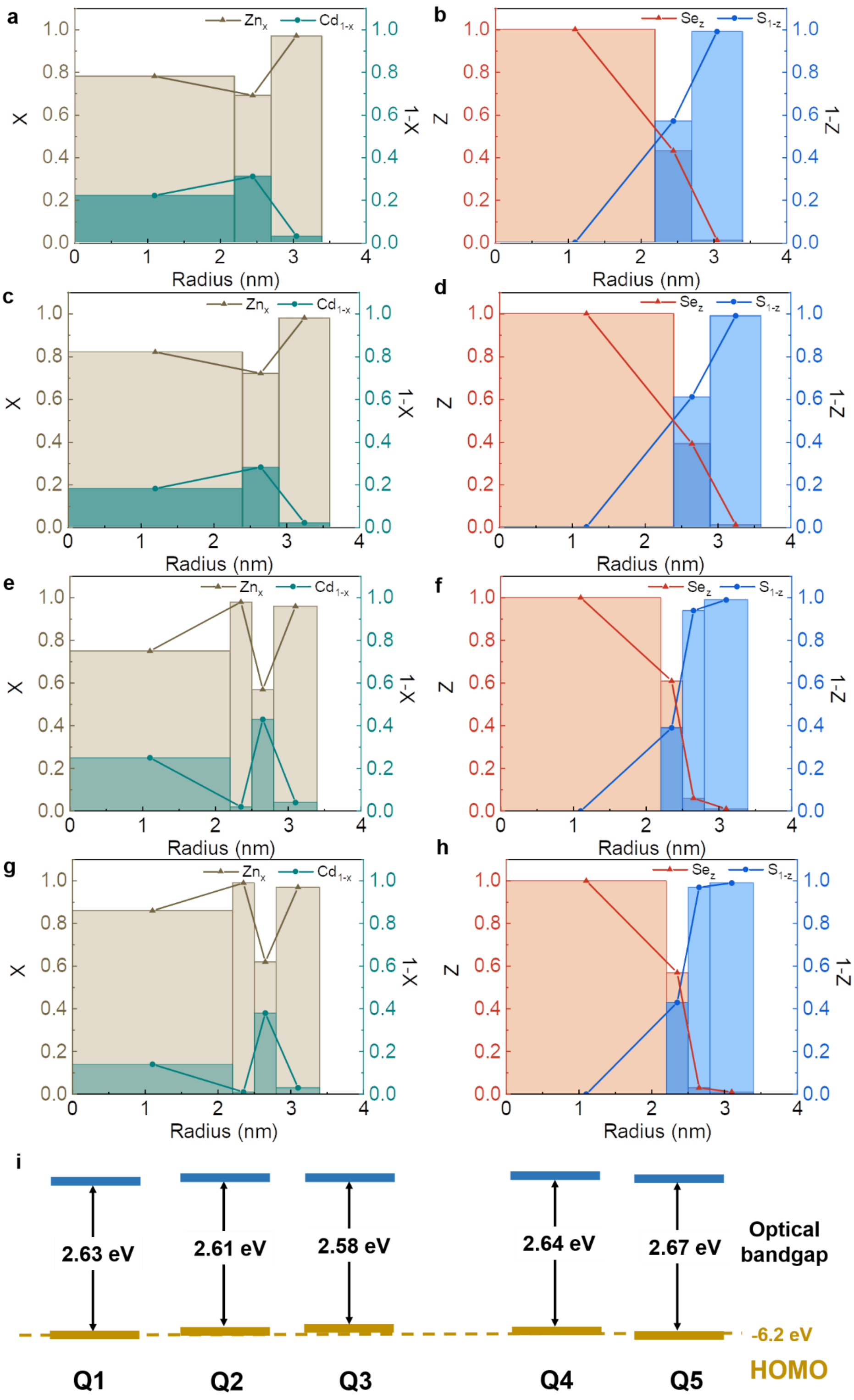

Supplementary Fig. 16 | Elemental and electronic structure of blue QDs. a and b, Radial distribution of cations and anions of Q2 ($Zn_xCd_{1-x}Se_z/Zn_xCd_{1-x}Se_zS_{1-z}/Zn_xS_{1-z}$). **c and d,** Q3 ($Zn_xCd_{1-x}Se_z/Zn_xCd_{1-x}Se_zS_{1-z}/Zn_xS_{1-z}$). **e and f,** Q4 ($Zn_xCd_{1-x}Se/ZnSe_zS_{1-z}/Zn_xCd_{1-x}S/ZnS$). **g and h,** Q5 ($Zn_xCd_{1-x}Se/ZnSe_zS_{1-z}/Zn_xCd_{1-x}S/ZnS$). The elemental ratios were measured using ICP-OES. The core/shell radii were measured using high-resolution TEM. **i,** The valance band maximum and optical bandgaps of Q1-Q5.

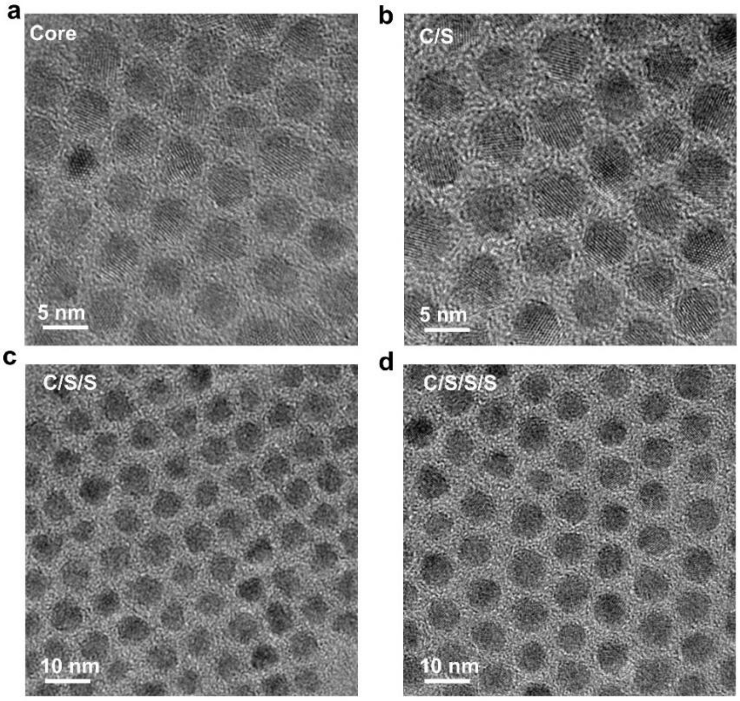

Supplementary Fig. 17 | TEM images of Q4. a-d, ZnCdSe (core), ZnCdSe/ZnSeS (C/S), ZnCdSe/ZnSeS/ZnCdS (C/S/S), and ZnCdSe/ZnSeS/ZnCdS/ZnS (C/S/S/S) of Q4, respectively.

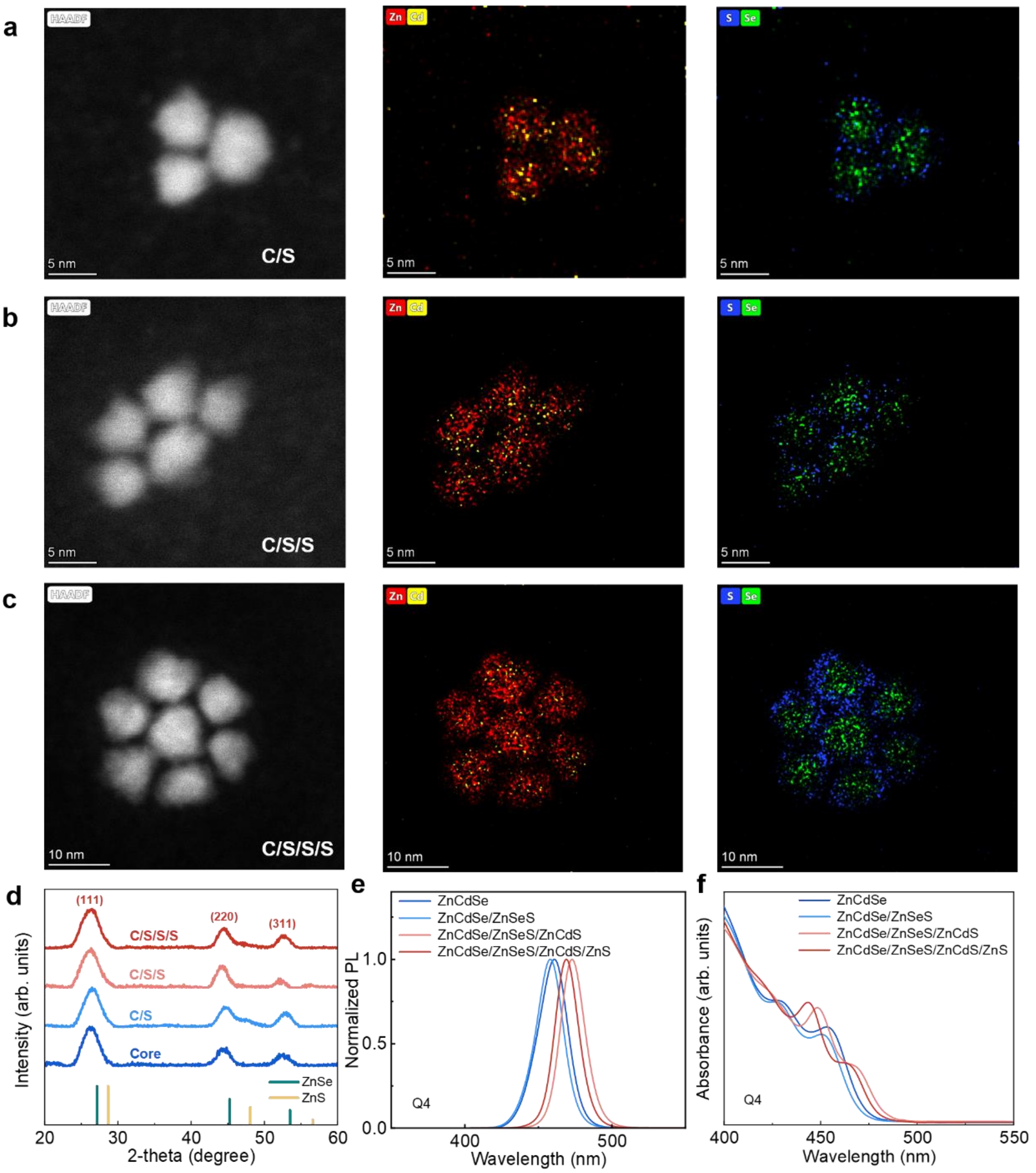

Supplementary Fig. 18 | Q4's C/S/S/S structure. a-c, HADDF-STEM images and the corresponding energy dispersive X-ray spectroscopy (EDX) elemental mapping. ZnCdSe/ZnSeS (C/S), ZnCdSe/ZnSeS/ZnCdS (C/S/S), ZnCdSe/ZnSeS/ZnCdS/ZnS (C/S/S/S) represent the samples after each growth stage of Q4. **d,** XRD patterns of core, C/S, C/S/S and C/S/S/S samples of Q4. The XRD patterns of bulk zincblende ZnSe and zincblende ZnS are provided as references. **e and f,** PL and absorbance spectra measured after each growth stage of Q4.

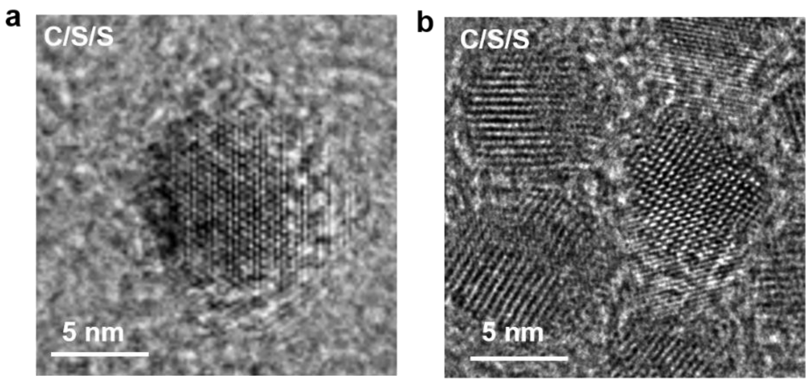

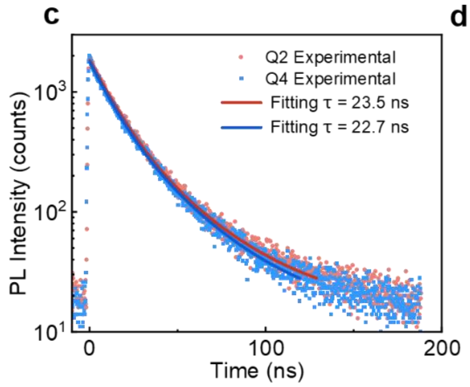

d

	A1	τ1	A2	τ2	τ
Q2	1385.39	13.09	451.51	35.36	23.51
Q4	1257.88	12.17	523.38	32.31	22.74

Supplementary Fig. 19 | Effect of nonmonotonically-graded shell on QDs' lattice and nonradiative recombination. a-b, High-resolution TEM images of the single Q2 and Q4, respectively. No noticeable lattice distortion is observed. **c,** Time-resolved PL spectra for Q2 and Q4. The solid lines are the fittings to the measured data, suggesting that the nonmonotonically-graded shell does not cause additional nonradiative recombination **d,** Fitting parameters for the PL decay.

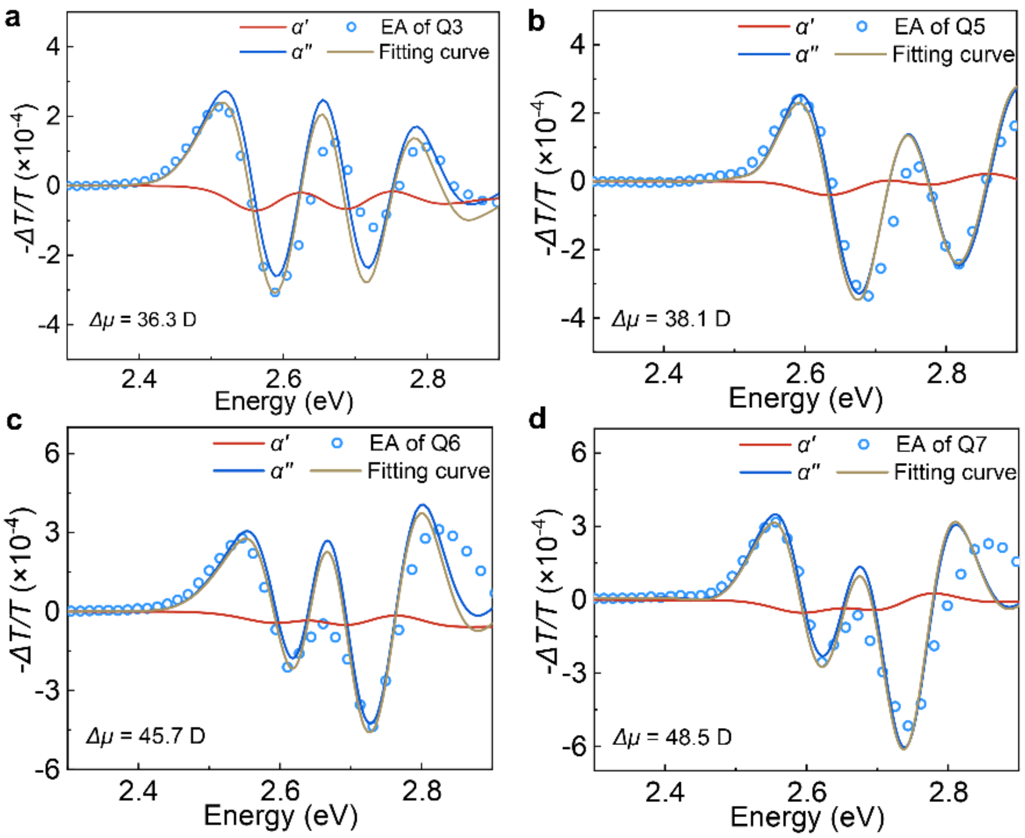

Supplementary Fig. 20 | Dipolar excited states of blue QDs. a-d, Electroabsorption spectra of the Q3-, Q5-, Q6-, and Q7-based samples with a structure of ITO/PEDOT:PSS/QDs/Ag(~15nm), respectively. The red and blue curves are *α'* and *α"* of QDs. The fitting curve is in dark yellow.

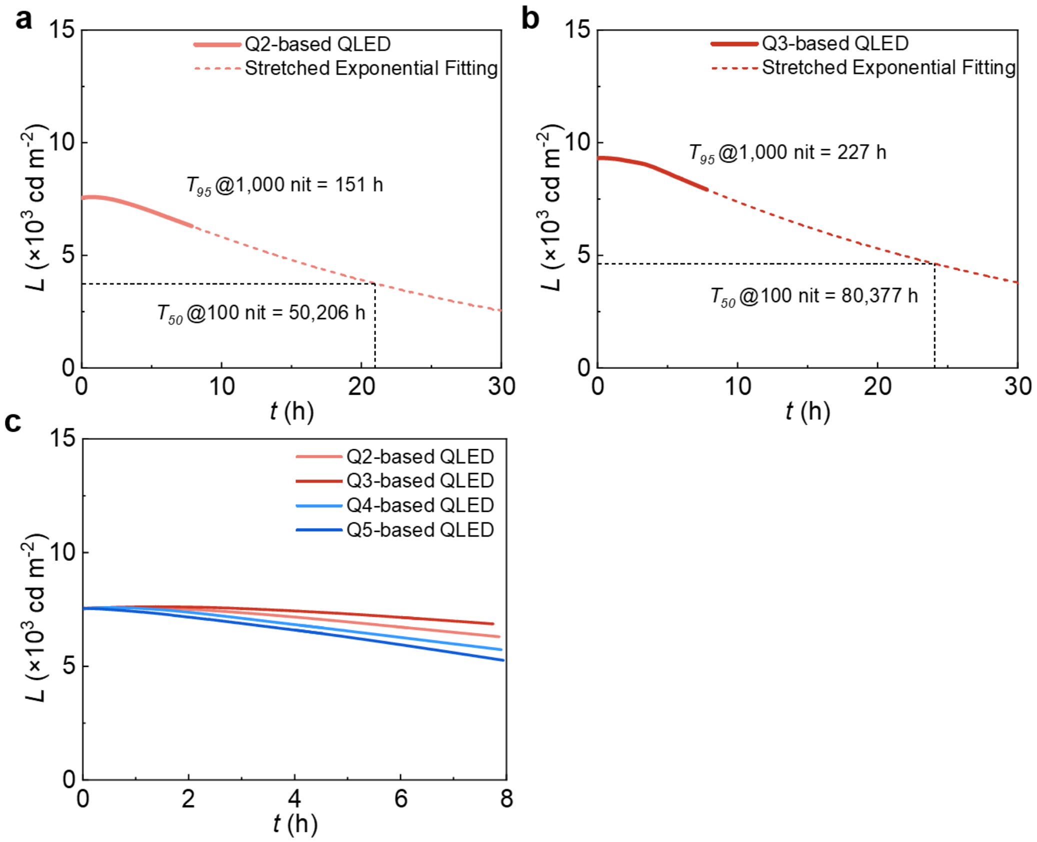

Supplementary Fig. 21 | Operational lifetimes. a and b, Operational lifetime of Q2- and Q3-based QLEDs. The extrapolated curves were fitted using stretched exponential decay functions[6,7] to obtain the T_{50} values. **c,** Operational lifetimes of Q2-, Q3-, Q4-, and Q5-based QLEDs under the same initial luminance.

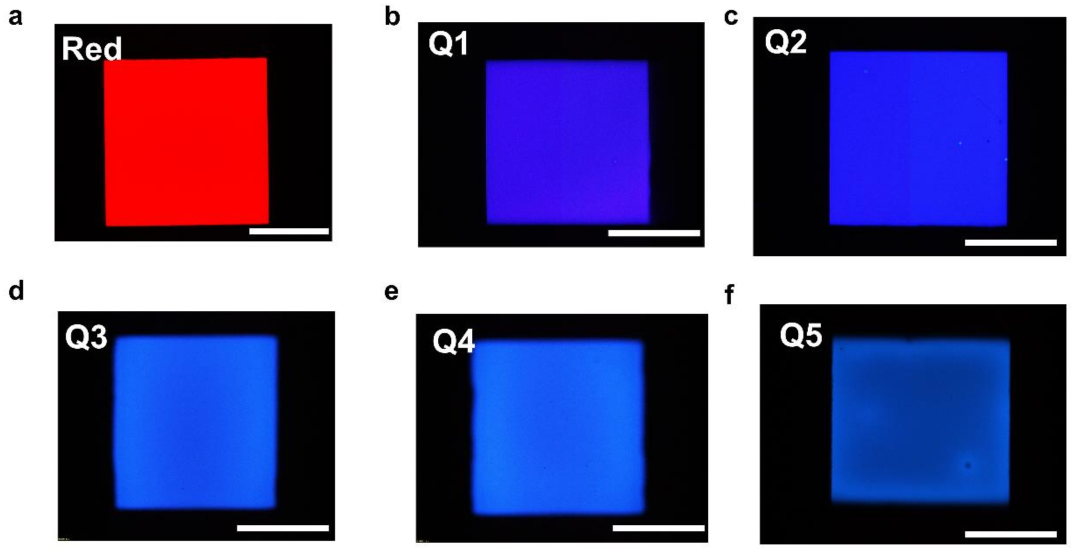

Supplementary Fig. 22 | Uniformity of luminance intensities. a-f, The microscopic image of corresponding red, Q1-, Q2-, Q3-, Q4-, and Q5-based QLEDs after lifetime test. The scale bar is 1 mm.

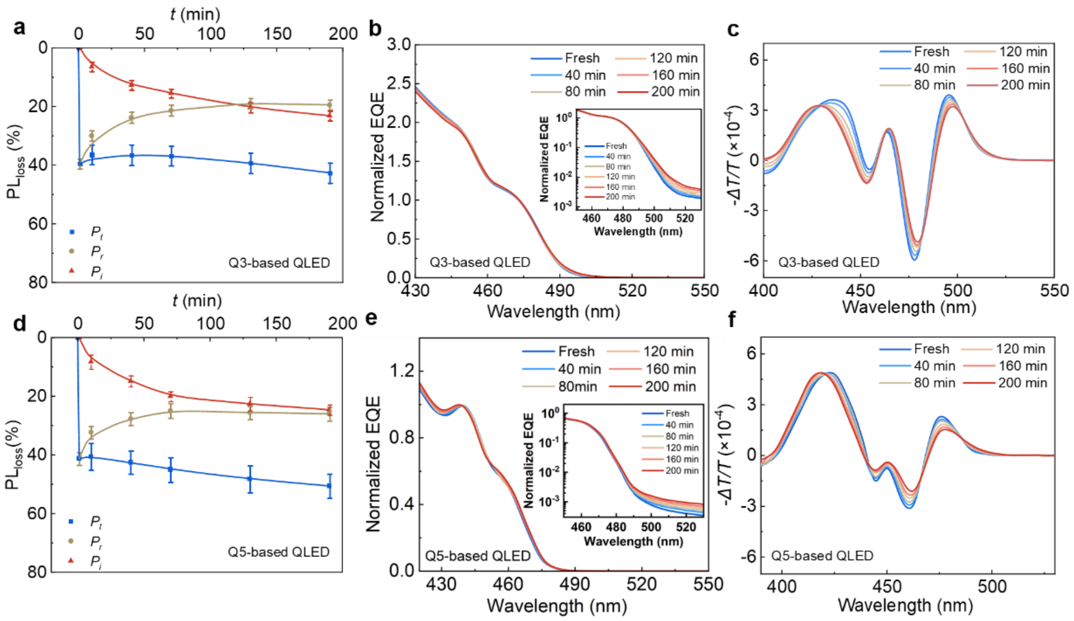

Supplementary Fig. 23 | EL-PL, sEQE and EA results of Q3- and Q5- based QLEDs. a and d, Reversible, irreversible and total PL loss of Q3 and Q5 measured from corresponding QLEDs as a function of device operation time. **b and e**, $sEQE_{PV}$ spectra of Q3- and Q5-based QLEDs. The inset shows the same $sEQE_{PV}$ plotted on a logarithmic scale for the demonstration of the near-band-edge transitions. **c and f**, Electroabsorption spectra of Q3- and Q5-based QLEDs measured as a function of operation time. All the tests are conducted with QLED devices driven by a constant current density of 100 mA cm^{-2}. The error bars in a and d mean the standard deviations calculated from the standard sample.

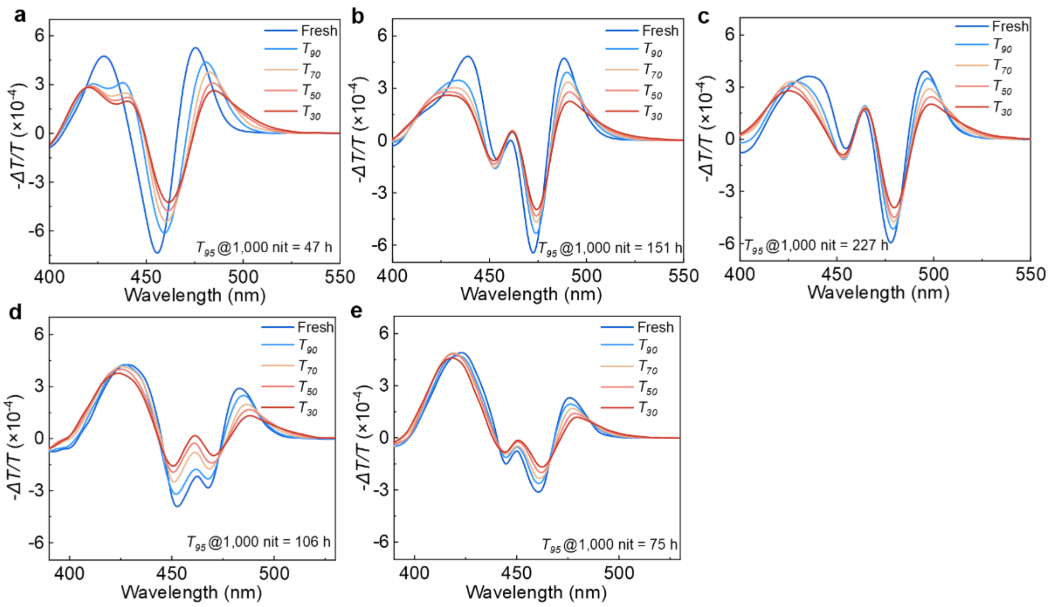

Supplementary Fig. 24 | EA lineshapes as a measure of the degree of device degradation. a-e, EA spectra of Q1-, Q2-, Q3-, Q4-, and Q5-based QLEDs, respectively. Measurements were taken when devices degraded to T_{90}, T_{70}, T_{50}, and T_{30}. Regardless the significant difference of operational lifetime, the three devices show very similar trend EA spectra change when degraded to the same degree.

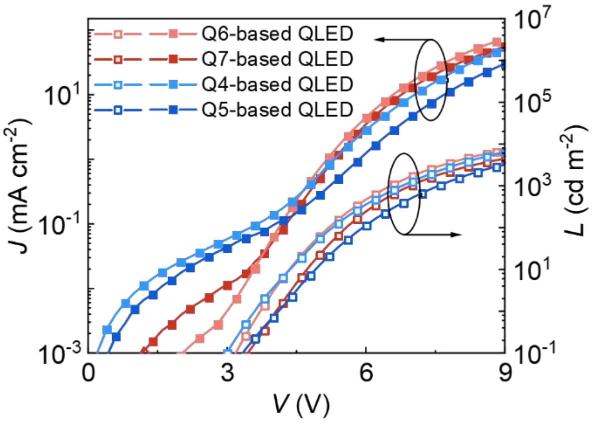

Supplementary Fig. 25 | *L*-*J*-*V* characteristics of Q4-, Q5-, Q6-, and Q7-based QLEDs.

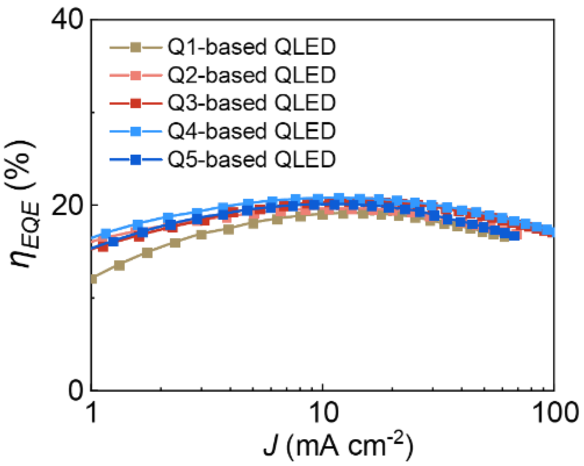

Supplementary Fig. 26 | Charge balance of Q1-, Q2-, Q3-, Q4-, and Q5-based QLEDs.

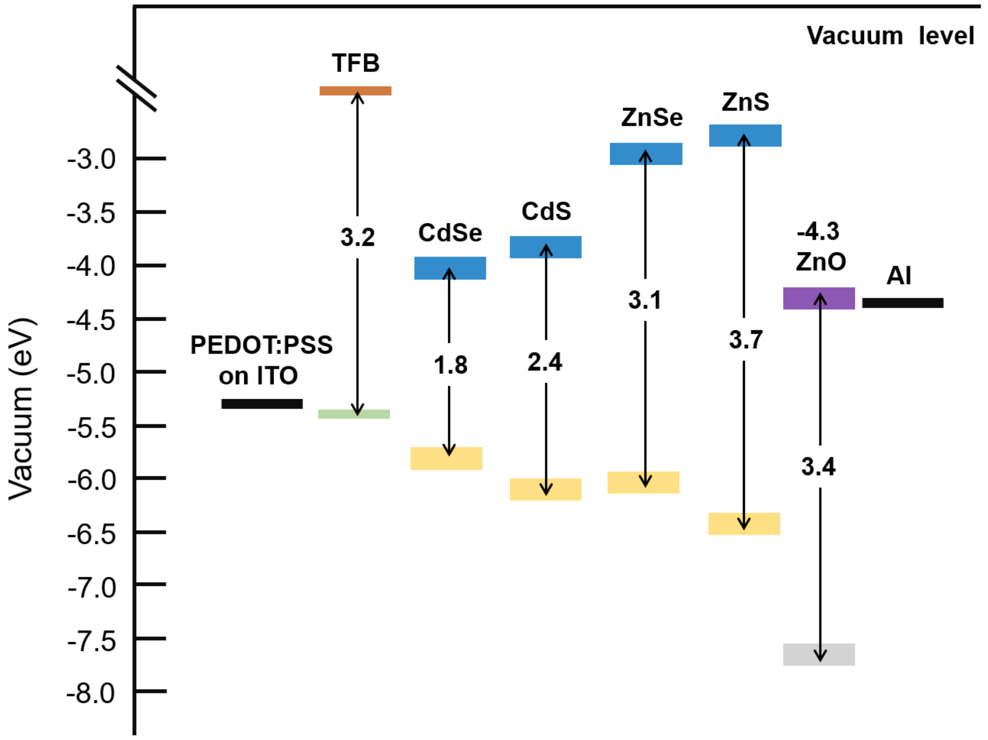

Supplementary Fig. 27 | Energy level diagram. The energy levels of electrodes, charge transporting materials, and II-VI materials used to compose gradient-alloyed QDs.

Solution-processed LEDs (sky blue)							
**Emitters**	**EL Peak (nm)**	**FWHM (nm)**	**CIE (x, y)**	**Max.EQE (%)**	**Lifetime (as reported)**	**$T_{95,re\text{-}calculate}$ (L_0=1000 cd m^{-2})**	**Reference**
CdZnSe/ZnS/ZnS QDs	479	34	(0.119, 0.154)	16.2	T_{50}@100 nit=355 h	5.5 h	ACS Photonics.(2018)[8]
ZnCdSe/ZnSe QDs	481	30*	(0.11,0.178)*	8.1	T_{50}@100 nit=7,000 h	9.6 h	Nat Photonics.(2019)[9]
CdSeS/ZnSeS/ZnS QDs	482	27*	(0.09,0.196)*	9.5*	T_{50}@100 nit>10,000 h	40 h	Nat Commun.(2020)[10]
CdZnSe/ZnS QDs	479	23	(0.1,0.157)	21.9	T_{50}@100 nit=24,000 h	57 h	Nat Photonics.(2022)[11]
$CsPbBr_3$ PeLED	478*	20	(0.091,0.144)*	12.3	T_{50}@90 nit=20 min	N/A	Nat. Nanotechnol.(2020)[12]
5CzCN OLED	479*	72*	(0.17, 0.31)	25.8	--	--	ACS A.M.I.(2018)[13]
ZnCdSe/ZnCdSeS/ZnS QDs	478	19	(0.112,0.125)	19.7	T_{50}@100 nit>50, 206 h	151 h	This work.(Q2)
ZnCdSe/ZnCdSeS/ZnS QDs	482	22	(0.098,0.177)	20.4	T_{50}@100 nit>80, 377 h	227 h	This work.(Q3)
**Solution-processed LEDs (deeper blue)**							
**Emitters**	**EL Peak (nm)**	**FWHM (nm)**	**CIE (x, y)**	**Max.EQE (%)**	**Lifetime (as reported)**	**$T_{95,re\text{-}calculate}$ (L_0=1000 cd m^{-2})**	**Reference**
ZnCdS/CdZnS/ZnS QDs	445	19	(0.155,0.022)*	18	T_{50}@100 nit=47.4 h	N/A	Nanoscale.(2018)[14]
ZnTeSe/ZnSe/ZnS QDs	460	35	(0.136,0.086)*	20.2	T_{50}@100 nit=15,850 h	30 h	Nature. (2020)[15]
ZnSe/ZnS QDs	445	<12	(0.161,0.014)*	12.2	T_{50}@100 nit=237 h	0.7 h	Nano Lett. (2021)[16]
CsPbBr3 PeLED	458	19*	(0.146,0.045)	1.1	--	--	Adv. Funct. Mater.(2021)[17]
CsPbBr3 PeLED	470	27	(0.13,0.11)	4.7	T_{50}@100 nit=12 h	N/A	Adv. Mater.(2021)[18]
CdZnS QDs	467	24*	(0.131,0.067)*	12.5	T_{50}@1,000 nit=23 h	3.6 h	Nat Commun.(2019)[19]
ZnCdSe/ZnCdSeS/ZnS QDs	474	21	(0.121,0.095)	19.2	T_{95}@1,000 nit=47 h	47 h	This work.(Q1)
ZnCdSe/ZnSeS/ZnCdS/ZnS QDs	471	20	(0.127,0.081)	20.8	T_{95}@1,000 nit=106 h	106 h	This work.(Q4)
ZnCdSe/ZnSeS/ZnCdS/ZnS QDs	467	20	(0.135,0.063)	20.1	T_{95}@1,000 nit=75 h	75 h	This work.(Q5)

Supplementary Table. 1 | Comparison of the solution-processed blue LEDs.

* indicates the data is extracted from published data graphs

N/A indicates the data cannot be obtained or recalculated from the provided data or graph.

— indicates the data was not provided

Supplementary References

1 Sebastian, L., Weiser, G. & Bässler, H. Charge transfer transitions in solid tetracene and pentacene studied by electroabsorption. *Chem. Phys.* **61**, 125-135 (1981).

2 Kazaoui, S. *et al.* Comprehensive analysis of intermolecular charge-transfer excited states in C 60 and C 70 films. *Physical Review B.* **58**, 7689 (1998).

3 Bernardo, B. *et al.* Delocalization and dielectric screening of charge transfer states in organic photovoltaic cells. *Nat Commun.* **5**, 3245 (2014).

4 Liu, T., Foo, Y., Zapien, J. A., Li, M. & Tsang, S.-W. A generalized Stark effect electromodulation model for extracting excitonic properties in organic semiconductors. *Nature communications.* **10**, 1-11 (2019).

5 Lane, P. A. Electromodulated doping of the hole transport layer in a small molecule organic light-emitting diode. *Journal of Photonics for Energy.* **1** (2011).

6 Féry, C., Racine, B., Vaufrey, D., Doyeux, H. & Cinà, S. Physical mechanism responsible for the stretched exponential decay behavior of aging organic light-emitting diodes. *Appl. Phys. Lett.* **87** (2005).

7 Scholz, S., Kondakov, D., Lussem, B. & Leo, K. Degradation Mechanisms and Reactions in Organic Light-Emitting Devices. *Chem. Rev.* **115**, 8449-8503 (2015).

8 Lin, Q. *et al.* Nonblinking Quantum-Dot-Based Blue Light-Emitting Diodes with High Efficiency and a Balanced Charge-Injection Process. *ACS Photonics.* **5**, 939-946 (2018).

9 Shen, H. *et al.* Visible quantum dot light-emitting diodes with simultaneous high brightness and efficiency. *Nature Photonics.* **13**, 192-197 (2019).

10 Pu, C. *et al.* Electrochemically-stable ligands bridge the photoluminescence-electroluminescence gap of quantum dots. *Nat Commun.* **11**, 937 (2020).

11 Deng, Y. *et al.* Solution-processed green and blue quantum-dot light-emitting diodes with eliminated charge leakage. *Nature Photonics.* (2022).

12 Dong, Y. *et al.* Bipolar-shell resurfacing for blue LEDs based on strongly confined perovskite quantum dots. *Nat Nanotechnol.* **15**, 668-674 (2020).

13 Jeon, S. K., Park, H. J. & Lee, J. Y. Highly Efficient Soluble Blue Delayed Fluorescent and Hyperfluorescent Organic Light-Emitting Diodes by Host Engineering. *ACS Appl Mater Interfaces.* **10**, 5700-5705 (2018).

14 Wang, O. *et al.* High-efficiency, deep blue ZnCdS/CdxZn1-xS/ZnS quantum-dot-light-emitting devices with an EQE exceeding 18. *Nanoscale.* **10**, 5650-5657 (2018).

15 Kim, T. *et al.* Efficient and stable blue quantum dot light-emitting diode. *Nature.* **586**, 385-389 (2020).

16 Gao, M. *et al.* Bulk-like ZnSe Quantum Dots Enabling Efficient Ultranarrow Blue Light-Emitting Diodes. *Nano Lett.* **21**, 7252-7260 (2021).

17 Zou, G. *et al.* Color-Stable Deep-Blue Perovskite Light-Emitting Diodes Based on Organotrichlorosilane Post-Treatment. *Adv. Funct. Mater.* **31** (2021).

18 Bi, C. *et al.* Perovskite Quantum Dots with Ultralow Trap Density by Acid Etching-Driven Ligand Exchange for High Luminance and Stable Pure-Blue Light-Emitting Diodes. *Adv. Mater.* **33**, e2006722 (2021).

19 Chen, S. *et al.* On the degradation mechanisms of quantum-dot light-emitting diodes. *Nature communications.* **10**, 1-9 (2019).
